# Global climate change and seasonal variation of cellulitis in hospitalized children: a 30 year retrospective study

**DOI:** 10.1017/S0950268825000032

**Published:** 2025-01-30

**Authors:** Orli Megged, Allon Raphael, Amalia Burstyn, Noy Deri, Shepard Schwartz, Rachel Eisenberg, Ori Toker

**Affiliations:** 1 Paediatric Department Paediatric Infectious Disease Unit, Shaare Zedek Medical Center, Faculty of Medicine, Hebrew University of Jerusalem, Israel; 2 Paediatric Department, Shaare Zedek Medical Center, Jerusalem, Israel; 3 The Hebrew University of Jerusalem, Hadassah Medical School, Israel; 4 Leumit Health Services, Modi’in-Illit, Israel; 5 Paediatric Department, Paediatric Allergy and Immunology Unit, Shaare Zedek Medical Center, Faculty of Medicine, Hebrew University of Jerusalem, Israel

**Keywords:** cellulitis, children, global climate change, seasonality

## Abstract

Cellulitis, a common subcutaneous infection, is influenced by host, pathogen, and environmental factors. Previous studies have shown seasonal patterns in adult cellulitis, suggesting temperature as a risk factor. This study investigated seasonal patterns in paediatric cellulitis in Jerusalem’s semi-arid climate. A single-center retrospective cohort study reviewed medical records of 2,219 hospitalized children under 18 with cellulitis between 1990 and 2020. Demographic, clinical, temperature, and humidity data were collected. Results revealed a significant sinusoidal pattern for limb cellulitis (LC) but for other body sites, with summer peaks and winter nadirs (*P* < 0.01). August showed the highest incidence, tripling that of February. Age groups 1-6 and 6-12 demonstrated the largest seasonal differences (*P* = 0.004, *P* = 0.008). Over three decades, paediatric hospitalized LC cases increased by 71% (*P* < 0.001), correlating with rising temperatures. Elevated ambient temperature seven days prior to diagnosis was a risk factor for LC development (OR = 1.02, *P* = 0.03). This study highlights the cyclic seasonal pattern of paediatric LC, peaking in summer. The significant increase in cases over time, coupled with rising temperatures, suggests climate change as a contributing factor. These findings could inform public health strategies for cellulitis prevention and management in children.

## Introduction

Cellulitis is a common infection in children and is associated with discomfort, erythema, swelling, and warmth of the affected area [1, 2]. Cellulitis that is accompanied by systemic symptoms or fails to respond to oral antibiotic therapy often requires hospitalization and intravenous antimicrobial therapy. [3, 4]

In adults, several risk factors have been demonstrated for cellulitis, such as older age, obesity, diabetes, and lymphedema [5–11]. These risk factors are less common in children, among whom common skin diseases such as atopic dermatitis or damage to the integrity of the skin, most commonly by trauma, have been linked to cellulitis [10–13].

Previous studies in adults have demonstrated a higher incidence of cellulitis during warmer months [7, 8, 14–20]. Two large studies from the United States reported that there were 35%–66% more cases of adult cellulitis in the month of July than in February [7, 8]. Although the risk factors of the Paediatric population are different from the adult population, only a few studies have included Paediatric patients in their cohorts [17, 18]. Additionally, to the best of our knowledge, only one study analyzed the correlation of daily weather data in the days preceding the diagnosis of cellulitis [19].

According to the European Environment Agency (EEA) and Intergovernmental Panel on Climate Change (IPCC), the global average surface temperature has increased by 0.74°C in the 20th century and there is a predicted average temperature rise of 1.5°C–5.8°C in the 21st century. Climate change involves both changes in temperature but also precipitation, wind and sunshine leading to shifts in seasonal patterns of infectious diseases. Climate change affects particularly vector-borne but also water-borne, air-borne and food-borne pathogens. Infectious diseases related to climate change/extreme weather include dengue, malaria, hantavirus and others such as cholera, salmonella, and giardia [21]. One example in Israel occurred in 2000 when a heatwave was associated with an outbreak of West Nile fever [22].

Jerusalem is located 31 degrees north and has a mixed subtropical semiarid climate with warm dry summers and cool rainy winters. Average daily maximum temperatures range from about 29 °C in July–August with average relative humidity of 40% to 11 °C in January with 57% humidity. The average annual precipitation is about 522 mm, mostly occurring between November to March (Israel Meteorological Service. From: https://ims.gov.il/he/ClimateAtlas).

In this study, we sought to determine the presence of seasonal patterns, analyze weather data in preceding days of illness, identify risk factors in children and lastly look at the effect of global climate change on the incidence of cellulitis in hospitalized children in Jerusalem, Israel, a region with a Middle Eastern climate. We demonstrate a seasonality to Paediatric limb cellulitis along with an overall rise in incidence over the past decades. We hypothesize that climate change, leading to a rise in ambient temperature, is a contributing factor to the rising incidence and seasonality of limb cellulitis. Additionally, we hypothesize that the seasonality of limb cellulitis could be explained by the different outdoor time spent and the type of clothing worn during the summer months.

## Materials and methods

### Clinical and demographic data collection

Computerized medical records of all Paediatric patients under the age of 18 years from 1990 to 2020, hospitalized for the treatment of cellulitis at Shaare Zedek Medical Center (SZMC) were reviewed. SZMC is a public 1000-bed facility, university and tertiary care medical centre that serves the population of Jerusalem, as well as the surrounding area, servicing about 1 million people with a high percentage of children ages 0–18 (42.8%) [23]. The International Classification of Diseases, 9th revision, Clinical Modification (ICD-9) codes for case ascertainment were used to stratify the study population into two groups: Limb cellulitis (LC) and Body cellulitis (BC). LC codes included 681 (cellulitis and abscess of finger and toe) and 682.3,4,6,7 (cellulitis and abscess of upper arm and forearm, hand except fingers and thumbs, leg except foot, foot except toes, respectively). BC codes included 682.0,1,2,5 (cellulitis and abscess of face, neck, trunk, and buttock, respectively). ICD code 682.8,9 (cellulitis and abscess of other specified or unspecified sites) were assigned to either the LC or BC group after review of the medical record. The following data were retrieved: age, sex, ethnicity, underlying atopic dermatitis (ICD code 691.8), molluscum contagiosum (ICD code 0.78), varicella infection (ICD code 052.9), body site of the cellulitis, date of admission and length of hospital stay.

### Meteorological data collection

Meteorological data, specifically maximum daily ambient temperature and humidity was collected from the Israel Meteorological Service (IMS.gov.il) and documented using Microsoft Excel 2003 (Microsoft, Redwood, WA). For every day in the 30-year period of research, we collected the maximum daily temperature and maximum daily humidity from the Jerusalem Center meteorological stations. The stations are located 3.5 km from SZMC at a height of 834 m above sea level similar to SZMC which is 830 m above sea level (Jerusalem height ranges from 570 to 857 m above sea level). Maximum daily temperatures and maximum daily humidity were collected for the seven days before the matched day of the patient’s admission.

Seasons were defined meteorologically based on temperature patterns and then equally divided into three-month periods [24]. Winter is December to February, the three coldest months in the Middle East; spring is March to May; summer is the three warmest months: June to August; and autumn is September to November.

### Statistical analysis

Continuous variables were expressed as a mean and 95% confidence interval (CI). Categorical variables were summarized as n (%) in each category. Comparison of continuous variables between two independent groups was done by Student’s t-test or Mann–Whitney as appropriate. Comparison of the distribution of two categorical variables was tested using the χ^2^ test and comparison of continuous variables between three independent groups was done by one-way ANOVA or Kruskal–Wallis as appropriate. Univariable and multivariable binary logistic regression were used to explore the risk factors. Any variable with *P* < 0.25 in univariate analysis was included in the model building of multivariable logistic regression. The odds ratio (OR) along with the 95% CI was reported. All statistical analyses were two-way and a *p-value* of 5% or less was considered statistically significant. The proportion of cases occurring in each season and each month was compared against an expected equal distribution by a one-way Chi-squared (goodness-of-fit) test. Temperature and humidity were compared by one-way ANOVA, Kruskal–Wallis test, and Spearman correlation to evaluate the correlation between the monthly counts of hospitalizations and the mean temperature and humidity. Statistical analysis was performed using IBM SPSS Statistics for Windows, Version 23.0. Armonk, NY: IBM Corp.

## Results

Two thousand two hundred and forty-four admissions with a primary diagnosis of cellulitis over the 30-year study period of hospitalized patients under the age of 18 were retrieved from the medical records. Twenty-five admissions (1%) were excluded due to incomplete data and twenty-seven (1%) were excluded due to readmission on the same or preceding month after discharge. Thus, 2,192 admissions were included in the study. The patient characteristics and results of the comparison of LC and BC are in Table 1. Overall, LC accounted for 1218(55%) cases in comparison to BC 1001(45%). Divided by age groups, LC was more common in the school-age groups: 66% in the elementary school age groups, ages 6–12, and 69% in the high school age, ages 12–18. In contrast, BC was more common in the preschool age: 51% in the pre-walking age, ages 0–1, and 55% in kindergarten age, ages 1–6 (*P* < 0.001). Cellulitis was more common in males in the whole study population (58% vs. 42%, *P* < 0.001) and in BC vs. LC (56% vs. 58% *P* = 0.42). Underlying skin conditions including atopic dermatitis, molluscum contagiosum, and varicella infection were not found to occur at different frequencies in LC or BC groups. (*P* = 0.54, 0.46 respectively).

**Table 1.** Demographic and clinical characteristics of the study population

	All patients n (%)	Limb cellulitis n (%)	Body cellulitis n (%)	P
	2,192	1,201 (55%)	991 (45%)	
Age (years, mean, 95% CI)	5.8[5.6–6.1]	6.7[6.4–7.0]	4.7[4.4–5.1]	<0.001* ^a^
Age groups (%)				
0–1 yr	392	195 (49)	197 (51)	<0.001* ^b^
1–6 yr	936	426 (45)	510 (55)	
6–12 yr	471	310 (66)	161 (34)	
12–18 yr	393	270 (68)	123 (32)	
Gender (%)				
Male	1,267	703 (55)	564 (45)	0.46 ^b^
Female	925	498 (53)	427 (47)	
Days of hospitalization				
mean [95% CI]	6.2[5.6–6.9]	6.9[5.8–8]	5.5[4.8–6.1]	0.01* ^c^
MC or AD (%)	53	27 (50)	26 (50)	0.57 ^b^
Varicella infection	32	12 (38)	20 (62)	0.48 ^b^

SD – standard deviation, CI – confidence interval, MC – Molluscum Contagiosum, AD – Atopic Dermatitis, LC – limb cellulitis, BC – body cellulitis,

* *P* < 0.05 indicate statistical significance.

a An independent samples t-test was conducted to compare mean of ages between BC and LC.

b A chi-square test was employed to evaluate the relationship between age groups, gender MC or AD and Varicella to BC and LC.

c The Mann–Whitney U test was employed to compare medians of days between the groups.

### Seasonality of cellulitis

**Figure 1.**
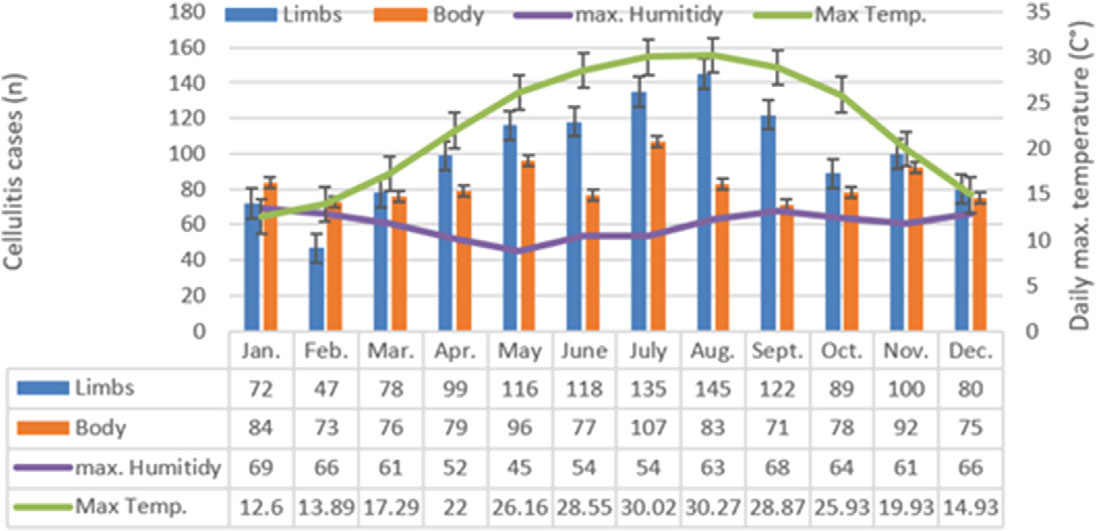
Seasonal distribution of LC and BC and ambient temperature and humidity.

The distribution of LC and BC by the four seasons and by months are represented in Table 2 and Figure 1, respectively. Over the study period 30% of all annual cases occurred during the summer, showing that the distribution of cases across seasons is not equal (*P* < 0.001) (Table 2). The distribution of cases across seasons was not equal in LC with the greatest gap between summer and winter (397(33%) vs. 193(16%), *P* < 0.01), more than a two-fold incidence of LC in the summer, in contrast to BC with a non-significant difference (264 (27%) vs. 242 (24%), *P* = 0.48). An annual seasonal pattern was found in the LC distribution, but not in the BC distribution (Figure 1). The total amount of LC cases requiring hospitalization in August was three times that of February (145 vs. 47), while the increment in July of BC was almost 1.5 times that in February (107 vs. 73) (Figure 1). Sub-analysis by age groups revealed that the greatest seasonal variation of LC was found among children 1–6 and 6–12 years of age, among whom the incidence in the summer was 36% and 34% while the winter accounted for only 14% of total cellulitis (*P* = 0.004, *P* = 0.008, respectively). Of the youngest age group, ages 0–1, there was no statistically significant difference in distribution between the four seasons (*P* = 0.27). When looking at mean temperatures a strong and significant positive correlation was found between monthly LC and mean temperature (R = 0.95, *P* < 0.001 (Figure 1)). A correlation was not found between LC and humidity (R = −0.56, *P* = 0.55). Additionally, a correlation was not found between BC and temperature or humidity (R = 0.3, *P* = 0.34, and R = -0.4, *P* = 0.16 respectively).

**Table 2.** Cellulitis by seasons and age groups and temperature and humidity by seasons

		Season				
	Total	Winter(%)	Spring(%)	Summer(%)	Autumn(%)	*P*
Total cellulitis	2,192	435 (20)	589 (27)	661 (30)	507 (23)	<0.001* ^a^
Limbs	1,201	193 (16)	335 (28)	397 (33)	276 (23)	
Body	991	242 (24)	254 (26)	264 (27)	231 (23)	
Age groups						
0–1 y						0.27 ^a^
LC		41(21)	57(29)	51(26)	46(24)	
BC		56(29)	54(27)	52(26)	35(18)	
1–6 y						0.004*
LC		61(14)	123(28)	154(36)	88(20)	
BC		114(22)	141(28)	142(28)	113(22)	
6–12y						0.008*
LC		44(14)	81(26)	107(34)	78(25)	
BC		42(26)	33(20)	43(27)	43(27)	
12–18 y						0.03*
LC		47(17)	74(27)	85(32)	64(24)	
BC		30(24)	26(21)	27(22)	40(33)	
Average daily meteorological parameters during 30 years of study (Mean ± SD)						
Max daily temperature (°C)		14 ± 4	24 ± 5	29 ± 2	21 ± 5	<0.001* ^b^
Daily relative humidity (%)		57 ± 21	37 ± 18	39 ± 11	47 ± 19	<0.001* ^b^

SD – standard deviation, LC – limb cellulitis, BC – body cellulitis,

* *P* < 0.05 indicate statistical significance.

a Chi-square test was performed to evaluate the association between cellulitis incidence and the four seasons.

b An analysis of variance (ANOVA) was employed to assess the differences in the average of daily maximum, temperature and daily relative humidity during the study period among the four seasons. Bonferroni-corrected post hoc tests were used for multiple comparisons.

### The effect over three decades

**Figure 2.**
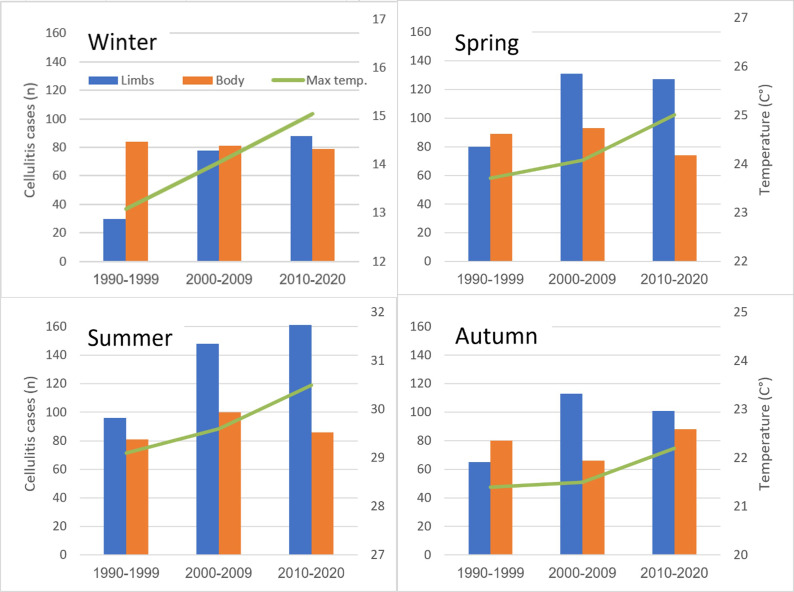
Incidence of body cellulitis and limbs cellulitis by decade. Average of seasonal max. daily temperature (green line) in Jerusalem by decade.

The incidence of cellulitis and average maximum daily temperature over three decades are represented in Table 3 and by seasons in Figure 2. Analysis of the three decades revealed an elevation of almost one degree (Celsius) in each season per decade. Additionally, the incidence of LC cases increased by 71% from 270 (45%) in the years 1990–1999 to 468 (60%) in the years 2010–2020 (*P* < 0.001) there were no changes in the incidence of BC cases (from 330 to 323, decreased by 2%, *P* = 0.84).

**Table 3.** Cellulitis and an average of max. Daily temperature over three decades

Decade	1990–1999 (%)	2000–2009 (%)	2010–2020 (%)	P
Total cellulitis	600 (27)	801 (37)	791 (36)	
LC	270(45)	463(58)	468(60)	<0.001* ^a^
BC	330(55)	338(42)	323(40)	
Max. daily temp. (°C, 95% CI)				
Winter	13.08 (12.8–13.3)	14.05 (13.7–14.3)	15.04 (14.7–15.3)	<0.001* ^b^
Spring	23.71 (23.3–24.1)	24.08 (23.7–24.4)	25.01 (24.6–25.3)	<0.001* ^b^
Summer	29.14 (28.9–29.3)	29.6 (29.4–29.7)	30.58 (30.4–30.7)	<0.001*
Autumn	21.4 (21.0–21.8)	21.5 (21.1–21.8)	22.2 (21.9–22.6)	0.005* ^b^

LC – limb cellulitis, BC – body cellulitis.

* *P* < 0.05 indicate statistical significance.

a chi square analysis compared LC vs. BC by three decades of study. Comparing the 2ed and 3ed dacaded *P* = 0.58.

b Kruskal Wallis test compared max. Daily temperature of each season separately by three decades of study.

### Multiple logistic regression

In order to check the effect of each factor on the incidence of LC, we performed a multivariate logistic regression analysis to control the effect of the clinical and demographic factors. The summarized regressions are represented in Supplementary Table S-1. We conducted a univariate analysis between the LC and the meteorological, demographic and clinical factors. The variables that were statistically significant in the univariate analysis (age groups, the summer season and maximum daily temperature 7 days prior to hospitalization, representing 2–3 days from skin wound to development of an infection and 2–4 days until an emergency room visit) were analyzed by multivariate analysis. In the multivariable analysis, we found that age groups 6–12 (OR = 1.9 (1.4–2.5) *P* < 0.001) and 12–18 (OR = 2.2(1.7–3.0), *P* < 0.001) and maximum daily temperature (OR = 1.03(1.01–1.05), *P* < 0.001) were independent risk for LC.

All factors were significant except gender, MC, AD, and varicella. We then used only the significant factors in the final model. The final model included 4 variables: age groups, maximum daily temperature and humidity in the 7 days prior to hospitalization (representing 2–3 days from skin wound to development of an infection and 2–4 days until an emergency room visit), and seasons. In the multivariable analysis we found that age groups 6–12 (OR = 2 (1.5–2.7) *P* < 0.001) and 12–18 (OR = 2.3(1.7–3.2), *P* < 0.001), maximum daily temperature (1.02(1.0–1.04), *P* = 0.03), humidity (1.007(1.001–1.01), *P* = 0.01) and the spring (OR = 1.4 (1.04–2.1) *P* = 0.02) were independent risk for LC.

## Discussion

In this study, we found that hospitalizations for cellulitis in children peaked during the summer months, along with an annual sinusoidal pattern in the incidence of LC. Previous studies have demonstrated conflicting results regarding the seasonal variability of cellulitis with some, including one from Israel, detecting an increase in erysipelas, superficial cellulitis and cellulitis cases during the summer months [7, 8, 14–20], while others failed to detect an association [25, 26]. Studies that did support a seasonal variability demonstrated up to a two-fold increase in adult cellulitis hospitalization in summer [6 and 20]. Our study demonstrated a threefold increase in hospitalization in summer peaks compared with winter nadirs in the Paediatric population, strengthening the literature by demonstrating both a clear sinusoidal pattern and an overall peaking incidence of cellulitis in the summer months. While several studies examined cellulitis inclusive of all parts of the body [6–8, 15–19] and others only examined lower extremity cellulitis [6, 14, 17, 20], We have separately examined limb cellulitis from other body parts, demonstrating that seasonality increased the incidence of limb cellulitis only. When looking at the correlation of temperature in the days prior to the diagnosis of cellulitis, the literature is sparse but tends to have a positive correlation. [17–19]. Our study adds to the sparsity of literature by demonstrating a positive correlation with rising temperatures in the days prior to the development of cellulitis.

Of concern, is the documented rise of 1 degree Celsius per decade over the last three decades along with a rising incidence of LC. The literature supports that with global climate change, there is an observed and expected rise in the prevalence of infectious disease [27]. When looking specifically at the change in the prevalence of cellulitis from decade to decade, studies from Australia support a rising prevalence [14]. Our study supports previous studies, by demonstrating that a rise in ambient temperature over the past three decades results in an increased incidence of cellulitis cases. Additionally, our study demonstrated that a 1-°C increase in temperature in the week prior to hospitalization is a risk factor for LC (OR = 1.02). This is in accordance with other studies showing an increase of 8°C being a risk factor for cellulitis with an OR of 1.33 [19]. Risk factors in the adult population for the above phenomenon include chronic venous insufficiency, lymphedema, obesity and leg ulcerations [6–12]. These risk factors do not apply to the Paediatric population. We hypothesized that defects in skin barrier that are commonly exacerbated in high temperatures such as atopic dermatitis, tinea pedis, and chronic carriers of staphylococcus aureus are likely risk factors in the Paediatric population [13, 28]. However, our study was not able to prove this correlation. Another explanation is that the higher incidence of cellulitis is not necessarily due to the heat but rather the indirect effects of heat on lifestyle. Specifically, during summer months children spend more time outdoors and are clothed more lightly thus exposing more skin to potential insect bites and accidental trauma, both of which are predisposing factors for cellulitis [11]. This may explain the statistically significant increased risk of LC and not BC. Additionally, this may also explain why this phenomenon was not seen in younger infants, under the age of 1, who are less mobile and more covered for them no seasonal changes of LC were found (*P* = 0.27) in contrast to other age groups. In accordance with previous reports [10] cellulitis was more common in males without any difference in BC vs. LC.

Limitations of this study include its retrospective nature and being conducted in a single medical centre reflecting local demographics. Additionally, the climate changes noted in our study may not necessarily apply to other populations and geographic areas. We were unable to evaluate the potential effect of the growth in the population and its effect on the incidence of cellulitis. The retrospective nature of this study did not enable us to collect sufficient data regarding other possible aetiologies such as insect bites, burns and local skin trauma. Concerning atopic dermatitis, data were collected, though the number of cases was low, therefore a potential correlation could not be assessed.

On the other hand, our study has several strengths: First, the length of our study (three decades) is longer than any previous studies therefore providing significantly more data and providing a more accurate analysis of seasonal trends, reflecting climate changes over extended time. Second, our study compared the incidence of limb vs. body cellulitis for the first time in the literature. Additionally, we studied specifically the Paediatric population, divided them into age groups, and were able to show risk factors in certain ages.

Future studies can focus on the Paediatric atopic population, defects of the skin barrier, and staphylococcus colonization, prospectively in order to determine risk factors for seasonal cellulitis development. Additionally, future research can evaluate Paediatric cellulitis in other climates as to whether the seasonal pattern of cellulitis holds true.

In conclusion, we found that Paediatric cellulitis requiring hospitalization has a seasonal pattern with a peak incidence during the summer, especially when involving the limbs of young children. We also found that with the rising temperatures over the past three decades, there is an overall increased prevalence. Mechanisms responsible for the seasonality of cellulitis are not yet fully understood, but minor trauma to exposed areas of skin because of outdoor activities and short clothes may play a role and may raise awareness for proper wound management. Knowledge of this seasonal variation and the effects of global climate change may improve our understanding of host-pathogen-environment interactions and help to improve public health surveillance by identifying potentially avoidable risk factors for cellulitis [29].

## Supporting information

Megged et al. supplementary material Megged et al. supplementary material

## Data Availability

The data is available from the corresponding author on reasonable request.

## Abbreviations

BC: Body cellulitis
LC: Limb cellulitis

## Supplementary material

For supplementary material accompanying this paper visit http://doi.org/10.1017/S0950268825000032. click here to view supplementary material
