# Supplementary material for: Global climate change and seasonal variation of cellulitis in hospitalized children: a 30 year retrospective study
Source: Epidemiol Infect. 2025 Jan 30;153:e24. doi: 10.1017/S0950268825000032 (PMC11869086; doi:10.1017/S0950268825000032)
Supplement: Megged et al. supplementary material [file S0950268825000032sup001.docx]

**Table S-1. Multivariable logistic regressions assessing risk for limb cellulitis by demographics and meteorological data. Dependent variable: LC**

|  | Univariate analysis | | Multivariate analysis | |
| --- | --- | --- | --- | --- |
|  | OR (95% CI) | P value | OR (95% CI) | P value |
| Gender | 1.06(0.9-1.2) | 0.44 |  |  |
| Age groups |  |  |  |  |
| 0-1y ^a^ |  |  |  |  |
| 1-6y | 0.5(0.4-0.6) | <0.001* | 0.8(0.6-1.1) | 0.1 |
| 6-12y | 1.7(1.4-2.2) | <0.001* | 1.9(1.4-2.5) | <0.001* |
| 12-18y | 2(1.6-2.5) | <0.001* | 2.2(1.6-3.0) | <0.001* |
| Varicella | 0.4(0.2-1) | 0.52 |  |  |
| MC or AD | 0.8(0.4-1.4) | 0.5 |  |  |
| temperature 7 days prior hospitalization | |  |  |  |
| Max. temp. ^b^ | 1.03(1.02-1.04) | <0.001* | 1.03(1.002-1.05) | <0.001* |
| Max humidity | 1(1.0 -1.01) | 0.09 |  |  |
| Seasons |  |  |  |  |
| Winter ^a^ |  |  |  |  |
| Spring | 1.2(0.9-1.3) | 0.23 |  |  |
| Summer | 1.3(1.1-1.6) | 0.001* | 1.01(0.8-1.3) | 0.9 |
| autumn | 0.98(0.8-1.1) | 0.85 |  |  |

OR -odds ratio, CI - confidence interval, MC - Molluscum Contagiosum, AD - Atopic Dermatitis, LC – limb cellulitis, BC – body cellulitis.

*p < 0.05 indicate statistical significance

^a^ Reference group

^b^ per 1˚C increase
